# Rap2a真核表达质粒的鉴定及其对肺癌细胞迁移能力的影响

**DOI:** 10.3779/j.issn.1009-3419.2014.09.01

**Published:** 2014-09-20

**Authors:** 金霞 吴, 苗苗 桑, 文嘉 曹, 骏年 郑, 冬生 裴

**Affiliations:** 1 221002 徐州，徐州医学院生理学教研室 Department of Physiology, Xuzhou Medical College, Xuzhou 221002, China; 2 221002 徐州，徐州医学院肿瘤生物治疗实验室 Research Institute of Cancer Prevention and Terapy, Xuzhou Medical College, Xuzhou 221002, China; 3 221002 徐州，徐州医学院附属医院临床肿瘤中心 Center of Clinical Oncology, Afliated Hospital of Xuzhou Medical College, Xuzhou 221002, China

**Keywords:** Rap2a, 基因克隆, 迁移, A549, H1299, Rap2a, Gene cloning, Migration, A549, H1299

## Abstract

**背景与目的:**

小G蛋白家族成员Rap2a可调控内皮素和细胞粘附从而影响细胞运动及细胞与基质间相互作用，但其在肿瘤发生发展中的作用仍属未知。克隆人Ras家族小G蛋白Rap2a的cDNA，构建其真核表达质粒并在肺癌细胞表达，初步探讨Rap2a在肺癌发生发展中的作用。

**方法:**

Western blot检测Rap2a在肺癌细胞中的内源性表达。人骨肉瘤细胞株U2OS提取细胞总RNA，经逆转录聚合酶链式反应逆转录成cDNA，PCR扩增*Rap2a*基因，酶切后插入pcDNA3.1(+)构建真核表达质粒pcDNA3.1(+)-Rap2a，采用酶切及测序鉴定。重组质粒转染H1299和A549细胞，Western blot检测目的基因表达。Transwell小室迁移实验观察Rap2a对肺癌细胞迁移能力的影响。明胶酶谱实验检测Rap2a对细胞分泌基质金属蛋白酶（matrix metalloproteinase, MMP）2的影响。

**结果:**

与正常细胞相比，肺癌细胞中Rap2a基础表达水平明显增高。双酶切及测序结果显示重组质粒pcDNA3.1(+)-Rap2a成功构建，Werstern blot检测到H1299和A549细胞有相应蛋白表达。迁移实验结果显示转染*Rap2a*基因后肿瘤细胞迁移能力明显增加。明胶酶谱实验结果显示Rap2a过表达后肺癌细胞分泌MMP2的量随之增加。

**结论:**

人Rap2a真核表达质粒成功构建，*Rap2a*基因在肺癌细胞株成功表达并能促进肺癌细胞的迁移能力。

## Abstract (en)

**Background and objective:**

Rap2a, a member of the small GTPase superfamily, plays a critical role in regulating the function of integrin and cell adhesion, thereby controlling cell motility and cell/matrix interactions. However, the function of Rap2a in carcinogenesis is still poorly understood. To clone Rap2a cDNA, which belongs to human Rasrelated small G protein superfamily, we constructed its eukaryotic expression vector and determined its expression in lung cancer cells. The aim of this study is to explore the role of Rap2a in carcinogenesis.

**Methods:**

The levels of endogenous Rap2a protein in lung cancer cells were measured by Western blot. Total RNA of human osteosarcoma cells U2OS was extracted and reverse-transcribed into cDNA by RT-PCR. Then, *Rap2a* gene was amplifed by PCR and inserted into pcDNA3.1(+). The reconstructed plasmid was identifed by restricted enzyme digestion and sequencing. pcDNA3.1(+)-Rap2a was transfected into H1299 and A549 cells, the expression of Rap2a was detected by Western blot. In addition, the migratory abilities of lung cancer cells were evaluated by Transwell assay. Matrix metalloproteinase (MMP)2 enzyme activity was evaluated by gelatin zymography.

**Results:**

Rap2a is signifcantly upregulated in lung cancer cells. The results of enzyme digestion and sequencing showed that the coding sequence of pcDNA3.1(+)-Rap2a was right and was inserted into the vector correctly. The results of Western blot showed that H1299 and A549 cells were transfected successfully. Transwell assay indicated that the ectopic expression of Rap2a promotes lung cancer cells migration. Correspondly, enzyme activity of MMP2 also increased.

**Conclusion:**

Eukaryotic expression plasmid pcDNA3.1(+)-Rap2a was constructed successfully. Rap2a could be expressed in lung cancer cells efciently and promotes lung cancer cell migration.

Ras超家族在信号转导过程中发挥着分子开关的作用，可作用于下游的多种受体蛋白，介导细胞外信号引起的胞质或胞核生物学特性的改变，调控细胞的多种生命活动进程^[1, 2]^。Rap蛋白(Rap1a, Rap1b, Rap2a, Rap2b, Rap2c)属Ras相关小GTP酶蛋白超家族。Rap1与Ras拥有相同的效应因子结构域，提示Rap1可与Ras竞争其下游效应因子，从而发挥拮抗Ras的功能。Rap2有其独特的生物学特性，其亚细胞定位主要集中在胞膜及内膜系统，在细胞静息状态下的活化水平超过50%，并且其效应因子结构域的第39位氨基酸残基为苯丙氨酸。因此，Rap2与Rap1和Ras在效应因子结构域的微小差异使得Rap2可能拥有特异的效应因子从而产生特定的生物学效应。

Rap2a最先于1988年由Pizon等^[3]^在对BurKitt's淋巴瘤cDNA文库进行筛选时被发现，主要定位于细胞膜和内膜系统。*Rap2a*基因全长549 bp，编码183个氨基酸，定位于13q34，其结构域组成与Ras家族蛋白相似，主要包括第32-40位氨基酸位置上的效应因子结构域，第11-148位氨基酸残基的5个散在分布的核苷酸结合域和C末端的CAAX基序。其C端CAAX基序与膜定位功能有关，此基序突变后可引起Rap2a许多功能丧失^[4, 5]^。值得一提的是，Rap在调节整联蛋白的功能和细胞粘附，进而控制细胞运动和细胞与基质的相互联系方面起着重要的作用^[6, 7]^。除了细胞这些生物学方面的基础性研究外，近些年来人们把目光又集中到Rap的一些特殊功能以及肿瘤方面的研究。肿瘤细胞的迁移和粘附在肿瘤侵袭转移中起着重要作用，有报道^[8, 9]^显示Rap2蛋白可影响人类多种肿瘤的迁移和粘附从而影响肿瘤的侵袭和转移。但其对肺癌的发生发展有何意义，目前尚未见报道。

## 1 材料和方法

### 1.1 材料

骨肉瘤细胞株U2OS，肺癌细胞株H1299和A549，人脐静脉内皮细胞(human umbilical vein endothelial cells, HUVEC)，DH5α感受态细胞及pcDNA3.1(+)为本实验室保存。限制性内切酶*Hin*dⅢ和*Eco*RI，RT-PCR试剂盒及T4 DNA连接酶购自Takara公司；PCR试剂盒购自天根生化科技有限公司；质粒DNA回收试剂盒和质粒小量提取试剂盒购自Promega公司；RNA抽提试剂盒、质粒中量提取试剂盒购自Qiagen公司；质粒转染试剂Lipofectamine^TM^ 2000为美国Invitrogen公司产品。兔抗人Rap2a抗体购自Abcam公司，抗人β-actin一抗购自美国Santa Cruz公司。荧光羊抗鼠和荧光羊抗兔IgG购自美国Licor公司，经Odyssey扫描仪扫描处理。

### 1.2 方法

#### 1.2.1 Western blot检测Rap2a在肺癌细胞中的表达

使用冰浴的细胞裂解buffer从各组细胞中提取细胞总蛋白。蛋白样品用12.5% SDS-PAGE跑胶后转膜至NC膜上。脱脂牛奶室温封闭1 h后用抗Rap2a抗体过夜。充分洗膜后室温孵育二抗1 h，再次洗膜后显影。

#### 1.2.2 cDNA的获取

人骨肉瘤细胞株U2OS弃去细胞表面培养基，胰酶消化细胞并用PBS冲洗后，按照Qiagen公司RNA抽提试剂盒说明书进行。抽提的RNA根据所测得的浓度取出相应含量的RNA用于反转录，获得cDNA文库。

#### 1.2.3 基因克隆

参照Genebank refseq中收录的NM_021033.6序列，设计针对*Rap2a*基因全长开放阅读框(open reading frame, ORF)区引物，上游5'GAAGCTTAT GCGCGAGTACAAAG3'，下游5'GGAATCCTATGTATG TACATG3'，以已获得的cDNA文库为模版，PCR扩增得到特异性产物。

#### 1.2.4 pcDNA3.1(+)-Rap2a质粒的构建

扩增的*Rap2a*基因，经*Eco*RI和*Hin*dⅢ双酶切后，用T4 DNA连接酶连接到同样用*Eco*RI和*Hin*dⅢ双酶切的pcDNA3.1(+)中，构建pcDNA3.1(+)-Rap2a真核表达质粒。经酶切初步鉴定后送上海华大基因科技有限公司测序验证。

#### 1.2.5 细胞转染

待细胞处于约80% - 90%融合时进行转染。按小皿的标准计算，每孔加入2 μg质粒，用Lipofectamine^TM^ 2000转染试剂进行转染。

#### 1.2.6 Transwell小室迁移实验

H1299和A549细胞转染Rap2a 24 h后，用胰酶消化收集细胞，用无血清培养基制成浓度为3×10^4^/mL的细胞悬液150 μL，加入Transwell小室的上室，下室加入600 μL含有10% FBS的条件培养基，继续在孵育箱中培养12 h。12 h后，室温固定，结晶紫染色，用湿棉签小心擦去上室底部膜表面上的细胞后显微镜下随机取5个视野拍照，计数并统计结果。

#### 1.2.7 明胶酶谱实验

细胞转染同前，转染后48 h换成1 mL无血清培养基继续培养24 h。次日收集上清液，将上清液移入离心管，2, 000 rpm离心10 min，BCA法测定各样品蛋白浓度，根据不同浓度确定样品的上样体积，保证各样品上样的蛋白质量相同，与4×上样缓冲液混合，跑胶，分别置于孵育液、洗脱液、脱色液中处理，显出基质金属蛋白酶(matrix metalloproteinase, MMP) 2位于蓝色背景上的透亮带，用凝胶图像分析系统分析读取条带面积，宽度和灰度值，做统计分析。

### 1.3 统计学方法

使用SPSS 16.0统计软件进行统计分析，实验数据以均数±标准差表示，两组间均数比较采用*t*检验分析。*P* < 0.05为差异有统计学意义。

## 2 结果

### 2.1 Rap2a蛋白在肺癌细胞中的表达水平

以HUVEC作为对照，采用Western blot技术检测Rap2a蛋白在H1299和A549两种肺癌细胞中的内源性表达量。结果显示相对于正常细胞组，肺癌细胞中Rap2a表达明显增高(图 1)。

**1:**
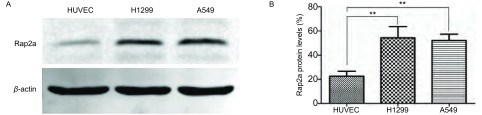
Rap2a蛋白在肺癌细胞H1299和A549中的表达。A：Western blot检测Rap2a蛋白在各组中的表达；B：Rap2a蛋白灰度分析。与HUVEC组比较：^**^P < 0.01(*n*=3). Rap2a protein expression in H1299 and A549 cell lines. A: Western blot was used to analyze the expression of Rap2a protein; B: Densitometric analysis of Rap2a. The intensity of Rap2a was quantified by densitometry (software: Image J, NIH). Compared with HUVEC group: ^**^P < 0.01 (*n*=3).

### 2.2 Rap2a真核表达质粒构建

以骨肉瘤细胞株U2OS抽提并反转录的cDNA为模板，用设计的Rap2a引物PCR，结果显示经琼脂糖凝胶电泳的PCR产物在500 bp-600 bp之间，与*Rap2a*基因的549 bp位置大概一致(图 2)。成功将*Rap2a*基因的全长ORF区克隆至pcDNA3.1(+)真核表达载体，用*Eco*RI和*Hin*dⅢ双酶切后经琼脂糖凝胶电泳验证并送专业公司测序，核酸测序结果与Genebank序列完全一致(图 3)。

**2:**
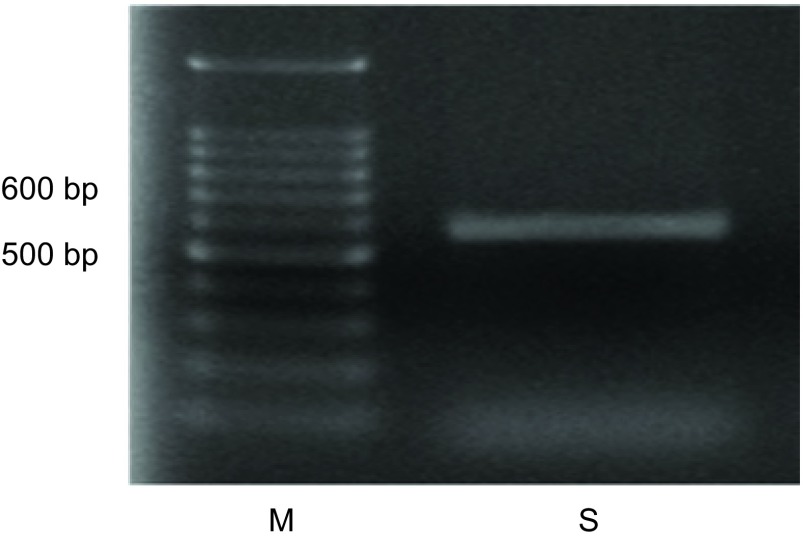
PCR鉴定Rap2a电泳图。M：1, 500 bp DNA Marker；S：Rap2a。 Rap2a of PCR amplification products. M: 1, 500 bp DNA Marker; S: Rap2a.

**3:**
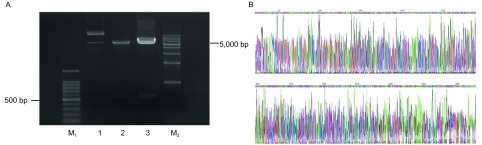
pcDNA3.1(+)-Rap2a克隆构建。A：由左至右依次为M_1_：1, 500 bp DNA Marker；1：pcDNA3.1(+)；2：pcDNA3.1(+)-Rap2a；3：pcDNA3.1(+)-Rap2a；M_2_：10, 000 bp DNA Marker；B：Rap2a测序结果峰图。 Vector construct of pcDNA3.1(+)-Rap2a. A is electrophoretogram of pcDNA3.1(+)-Rap2a, from left to right: DNA Marker, pcDNA3.1, pcDNA3.1(+)-Rap2a, pcDNA3.1(+)-Rap2a, DNA Marker; B is sequencing result of Rap2a.

### 2.3 pcDNA3.1(+)-Rap2a在真核细胞中表达的鉴定

为进一步检测Rap2a蛋白是否表达成功，我们用pcDNA3.1(+)-Rap2a转染H1299和A549细胞，提取总蛋白，用抗Rap2a抗体检测Rap2a在H1299及A549细胞的表达，以转染pcDNA3.1(+)空质粒组作为对照。结果显示相对于空白载体组，过表达组Rap2a表达明显增加(图 4)。

**4:**
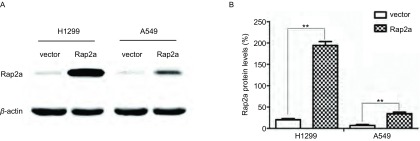
转染Rap2a质粒后Western blot检测Rap2a蛋白的表达。A：Western blot检测Rap2a过表达效果；B：Rap2a蛋白灰度分析。与vector组比较：^**^P < 0.01 (*n*=3). Western blot analyses of Rap2a after transfection with the Rap2a expressing vector. A: Western blot was used to analyze the expression of Rap2a protein after transfection with Rap2a; B: Densitometric analysis of Rap2a. The intensity of Rap2a was quantified by densitometry (software: Image J, NIH). Compared with vector group: ^**^P < 0.01 (*n*=3).

### 2.4 Rap2a对肿瘤细胞迁移能力的影响

我们对两种肺癌细胞进行了Transwell细胞迁移实验。用pcDNA3.1(+)-Rap2a转染H1299和A549细胞，以转染pcDNA3.1(+)空质粒组作为对照。结果显示实验组细胞的迁移率明显高于空白载体组，说明Rap2a过表达可促进肺癌细胞H1299和A549的迁移(图 5)。

**5:**
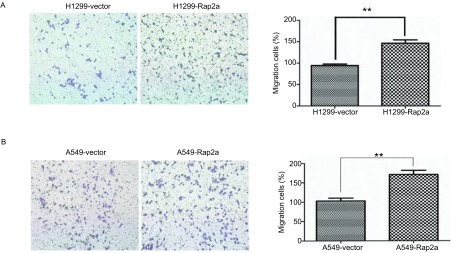
Rap2a过表达后对肺癌细胞H1299和A549迁移能力的影响（×100）。A：Transwell检测细胞迁移能力。B：细胞穿膜细胞数统计图。与vector组比较：^**^P < 0.01 (*n*=3). The impact of Rap2a overexpression on lung cancer cell migration (×100). A: Transwell were applied to test the impact of Rap2a expression on the cell migration of H1299 and A549 after transfection; B: Statistical figure of the numbers of cell permeating septum. Compared with vector group: ^**^P < 0.01 (*n*=3).

### 2.5 Rap2a过表达后对细胞分泌基质金属蛋白酶MMP2的影响

明胶酶谱实验检测了H1299和A549两种肺癌细胞分泌MMP2活性的表达。结果显示，Rap2a过表达后两种肺癌细胞分泌MMP2的量明显增加。灰度分析其MMP2表达量也明显增加，差异有统计学意义(*P* < 0.01)。说明上调Rap2a的同时也能上调MMP2的表达(图 6)。

**6:**
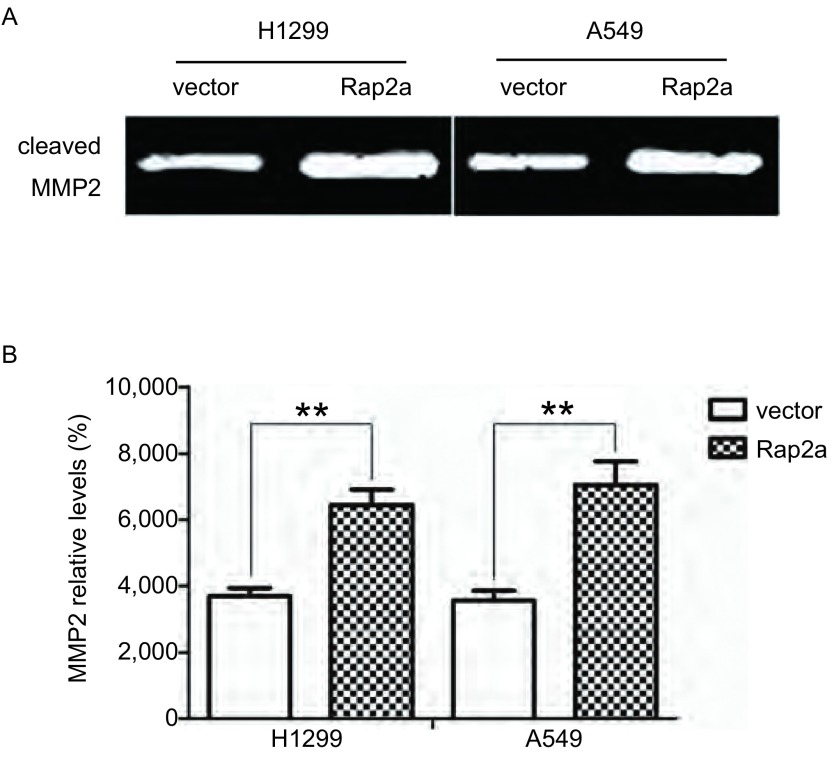
Rap2a过表达后明胶酶谱检测细胞分泌MMP2的情况。A：明胶酶谱检测细胞分泌MMP2情况；B：MMP2灰度分析。与vector组比较：**P < 0.01 (*n*=3). Gelatin zymography analysis of the relative enzyme activities of cleaved-MMP2 after overexpression of Rap2a. A: Gelatin zymography was used to analyze the expression of MMP2 after transfection with Rap2a; B: Densitometric analysis of MMP2. The intensity of MMP2 was quantified by densitometry (software: Image J, NIH). Compared with vector group: **P < 0.01 (*n*=3). MMP: matrix metalloproteinase.

## 3 讨论

肿瘤的发生来自于体细胞突变后产生致癌基因或抑制了肿瘤抑制基因，它是一个多步骤的过程。肿瘤细胞的两大特点是失控性的增殖和侵袭^[10]^。Rap蛋白是一个小分子量GTP结合蛋白家族，与*Ras*原癌基因具大多数序列同源性^[11]^。自从Rap家族被发现，人们将大多的注意力集中在Rap1蛋白。有研究结果显示Rap1的异常活化能促进肿瘤细胞的增殖和侵袭^[12, 13]^，且Rap1GAP能抑制细胞骨架的重构和甲状腺癌细胞的运动^[14]^。最近有报道^[7]^显示Rap2蛋白与Rap1类似，也能调节细胞的粘附。Rap1GAP和Rap2共表达在正常的甲状腺滤泡细胞。在正常的甲状腺细胞中无Rap1表达，但是Rap1却出现在乳头状甲状腺癌中，而且甲状腺癌中也发现了Rap2的高表达。这表明Rap活性的增强与肿瘤发生紧密相关。两种Rap蛋白的出现提示当Rap1GAP下调后Rap1和Rap2都被活化。当低水平Rap1GAP瞬时表达，其能力可充分阻断乳头状或未分化甲状腺癌细胞株的内源性Rap2活性时，Rap1GAP可以减弱细胞迁移侵袭的能力^[15]^。Dominissini等^[16]^也发现Rap2a和AMFR通过下调microRNA成为胶质瘤迁移和侵袭的主要调节者。Prabakaran等^[17]^发现从滤泡甲状腺癌分离的侵袭性细胞Rap2a高表达。而且，有报道^[18]^显示Rap2与肿瘤的形成及恶性转变也有关，Rap2下游效应因子RPIP9在乳腺癌中活性升高，而且其活性与乳腺癌的恶性转移程度密切相关。而Rap2另一下游效应因子MAP4K4在多种肿瘤组织中高表达，其活性程度与肿瘤的侵袭及转移相关^[19]^。但是，Bigler等^[20]^却发现前列腺癌细胞中活化的Rap2a下降。综上所述，Rap2a对肿瘤细胞的迁移能力是促进还是抑制作用目前还存在一定争议，且其对肺癌细胞迁移能力的影响未见报道。

本实验克隆得到了*Rap2a*基因的全长ORF区的真核表达质粒，通过转染H1299和A549两种肺癌细胞发现Rap2a蛋白成功表达于肺癌细胞。通过Transwell小室迁移实验发现，Rap2a过表达后能引起肺癌细胞迁移能力增强，且作用效果明显。MMP2是一种基质金属蛋白酶，属MMPs家族，几乎能降解细胞外基质中各种蛋白，在肿瘤侵润中发挥重要作用。明胶酶谱实验结果显示Rap2a过表达使细胞分泌MMP2的量随之增加。这些结果提示*Rap2a*很可能成为新的候选癌基因。本研究将为后续研究Rap2a对肿瘤细胞的其它功能打下基础，在后期研究中我们将致力于扩展*Rap2a*基因在肺癌细胞中的功能和机制研究及体外成瘤实验，为肺癌的早期诊断和治疗提供一定理论依据。
