# 更正声明

**DOI:** 10.3779/j.issn.1009-3419.2021.101.07

**Published:** 2021-03-20

本刊2014年第17卷第9期刊登的题为“Rap2a真核表达质粒的鉴定及其对肺癌细胞迁移能力的影响”[吴金霞, 桑苗苗, 曹文嘉, 等. Rap2a真核表达质粒的鉴定及其对肺癌细胞迁移能力的影响. 中国肺癌杂志, 2014, 17(9): 643-648.]一文中：第645页图 1和第647页图 6因作者失误导致图片错误，希望修正如下图。特此更正。对此深表歉意。

Erratum: Identification Analysis of Eukaryotic Expression Plasmid Rap2a and Its Effect on the Migration of Lung Cancer Cells

Jinxia WU, Miaomiao SANG, Wenjia CAO, *et al*.

Zhongguo Fei Ai Za Zhi, 2014, 17(9): 643-648.

In the version of this article initially published, error appeared in Fig 1 on page 645 and Fig 6 on page 647. Due to the author’s figure errors, the authors expect to make corrections and the correct figures are as below:

**1:**
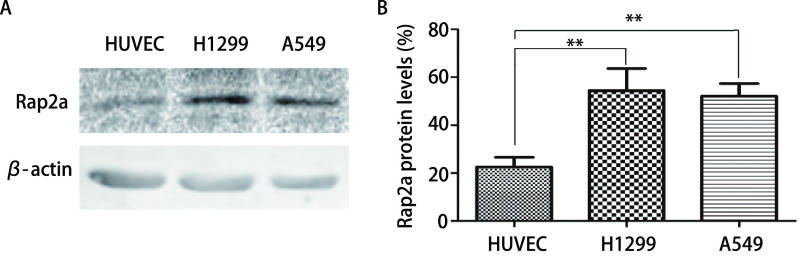
Rap2a蛋白在肺癌细胞H1299和A549中的表达。A：Western blot检测Rap2a蛋白在各组中的表达；B：Rap2a蛋白灰度分析。与HUVEC组比较：^**^P<0.01 (*n*=3). Rap2a protein expression in H1299 and A549 cell lines. A: Western blot was used to analyze the expression of Rap2a protein; B: Densitometric analysis of Rap2a. The intensity of Rap2a was quantified by densitometry (software: Image J, NIH). Compared with HUVEC group: ^**^P<0.01 (*n*=3).

**6:**
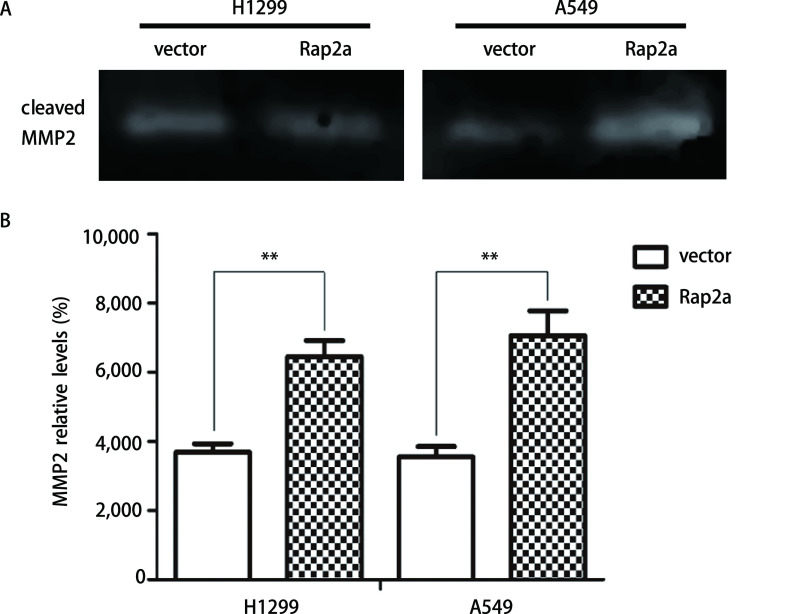
Rap2a过表达后明胶酶谱检测细胞分泌MMP2的情况。A：明胶酶谱检测细胞分泌MMP2情况；B：MMP2灰度分析。与vector组比较：^**^P<0.01 (*n*=3). Gelatin zymography analysis of the relative enzyme activities of cleaved-MMP2 after overexpression of Rap2a. A: Gelatin zymography was used to analyze the expression of MMP2 after transfection with Rap2a; B: Densitometric analysis of MMP2. The intensity of MMP2 was quantified by densitometry (software: Image J, NIH). Compared with vector group: ^**^P<0.01 (*n*=3). MMP: matrix metalloproteinase.

## Notes

https://www.ncbi.nlm.nih.gov/pmc/articles/PMC6000507

